# Detectability and Persistence of *Cyclospora cayetanensis* Oocysts in Artificially Contaminated Soil and Fresh Herbs Grown Under Controlled Climatic Conditions

**DOI:** 10.3390/pathogens14050430

**Published:** 2025-04-28

**Authors:** Ellie L. Rogers, Joseph Arida, John Grocholl, Joyce Njoroge, Sonia Almeria

**Affiliations:** 1 Joint Institute for Food Safety and Applied Nutrition (JIFSAN), University of Maryland, College Park, MD 20742, USA; ellielaurenrogers@gmail.com (E.L.R.); joseph3ali@gmail.com (J.A.); 2 Virology and Parasitology Branch, Division of Food and Environmental Safety, Office of Applied Microbiology and Technology, Office of Laboratory Operations and Applied Sciences, Human Food Program (HFP), Food and Drug Administration (FDA), Laurel, MD 20708, USA; grocholljohn@gmail.com (J.G.); joyce.njoroge@fda.hhs.gov (J.N.)

**Keywords:** protozoa parasite, sandy clay loam, silt loam, basil, parsley, cilantro, growth chambers, presence

## Abstract

*Cyclospora* oocysts are thought to be highly resistant in the environment but the climatic factors which determine the presence/persistence of *Cyclospora* oocysts are currently unknown. The main objective of this study was to determine the effects of temperature, water content, and soil texture on *C. cayetanensis* detection/persistence in artificially contaminated soil and herbs grown under controlled environmental conditions. Soil and leaves of three potted herbs (cilantro, parsley, and basil) grown in growth chambers and inoculated with *C. cayetanensis* oocysts were collected at 7, 14, 21, 28–31, 35–38, 42–45, 49–52, and 56 days post inoculation (dpi). Under wet watering conditions, independent of temperature, positive *C. cayetanensis* detection was observed at each sampling collection in both soil and herb leaves. Additionally, all three herbs were found to be positive for the parasite throughout the study duration in arid watering conditions. Conversely, short-lived persistence in soil was observed under arid conditions independent of temperature in Sandy Clay Loam soil (up to 14 dpi) and in Silt Loam soil (up to 21 dpi). Our results on the effect of desiccation on the presence and persistence of oocysts may provide useful insights for the proper cleaning and sanitizing of utensils or food contact surfaces to help control the persistence of the parasite.

## 1. Introduction

*Cyclospora cayetanensis* is a worldwide foodborne human parasite which causes a diarrheal illness called cyclosporiasis [1,2,3]. In endemic developing areas, the most susceptible populations are children, newcomers, and immunocompromised patients. In industrialized/developed countries, where *C. cayetanensis* infection has been related to traveler’s diarrhea and to the consumption of contaminated fresh produce mainly from endemic regions, infection affects people of any age [3].

Outbreaks of cyclosporiasis have affected thousands of persons and have been mainly associated with the consumption of fresh produce such as herbs, berries, snow peas, and mixed salads, among others [3,4,5,6,7,8,9]. In North America, cyclosporiasis outbreaks have occurred every year since the 1990s in a multi-state fashion [3,4,10,11,12,13]. In 2023, according to the latest surveillance data in the US, 2272 domestically acquired cases of cyclosporiasis were reported across 40 states [14]. Importantly, although in the past outbreaks in the US were mostly associated with imported fresh produce or international travel, recently, the parasite has been detected in domestically grown cilantro and Romaine lettuce [15]. Precise figures on the total economic burden of cyclosporiasis are difficult to obtain due to the underreported nature of cases and challenges in accurately attributing outbreaks to specific food sources [16].

Those infected with *C. cayetanensis* release unsporulated oocysts in their feces which may contaminate the environment. Under appropriate and still unknown climatological conditions which may vary by climatic region [3], the oocysts sporulate and become infectious. Consumption of food or water with sporulated oocysts can cause new infections. As such, direct person–person transmission is unlikely [3]. Under laboratory conditions, oocysts stored in deionized water or potassium dichromate at 22 °C and 30 °C can sporulate in 5–14 days [17,18]. In their infectious stage, a sporulated *C. cayetanensis* oocyst contains two sporocysts, each with two sporozoites [17,18]. Sporozoites are the infectious stage of the parasite which initiate the parasitic cycle when they multiply in the human intestinal tract. *Cyclospora cayetanensis* oocysts can contaminate fresh produce through contaminated agricultural water, by irrigation or by spraying of crops with wastewater (black water) [19]. The inadequate defecation disposal and contamination of soils might be a significant determinant for infection, as well as infected food handlers, or hands that have been in contact with contaminated soil [19]. Higher rates of infection have been noted in areas where risk factors such as deficient sanitary facilities, poor personal hygiene, and soil contaminated with human feces were present in some agricultural settings [20,21,22].

Protozoa oocysts, including those of *C. cayetanensis*, are environmentally resistant and are expected to remain infective in the environment for extended periods of time. The high resistance of protozoa, including *C. cayetanensis*, in the environment is mediated by the morphology of their cyst/oocyst, which involves a highly resistant cell wall that allows for survival at adverse environmental conditions, as well as conventional disinfection processes [23,24]. Oocysts do not multiply in the environment or in food, nor do they have a direct effect on growing plants, but they do play a critical role in the parasitic transmission cycle. In general, most protozoa oocysts require a minimum amount of time, moisture, and moderate temperature to sporulate and become infective. Furthermore, it has been established that humidity helps to facilitate both the development and transmission of coccidian oocysts [25]. However, different parasites may respond differently to variations in environmental conditions. For example, under conditions of severe desiccation, *Eimeria* oocysts remain viable for a significantly longer period than the *Cryptosporidium* oocysts [26]. At present, there are no animal models, and/or in vivo and in vitro culture systems to study the survival and/or viability of environmental *C. cayetanensis* oocysts. Data regarding long-term survival are available from other morphologically similar apicomplexan protozoa for which there are methods to assess viability. For example, extreme hardiness and long-time duration in the environment has been reported for *Toxoplasma gondii* oocysts. *Toxoplasma* oocysts have similar size and double wall oocysts as those of *C. cayetanensis*. *Toxoplasma* oocysts can survive outdoors in soil buried at a depth of 3–9 cm for 18 months in dry weather conditions in Texas [27] and have been reported to survive and remain infective for years in fresh water [28] and for at least twenty-four months in salt water [29]. Drying under low humidity and high temperature has been shown to be deleterious for *T. gondii* oocysts [30]; likewise, being inactivated when exposed to temperatures over 60 °C (cooking). Ultraviolet rays, depending on the dose, have also shown deleterious effects on oocysts viability [30]. Similarly for *Cryptosporidium*, another important foodborne and waterborne protozoan, oocysts can survive for months in soil and water [31], and for at least a year in seawater [32]. However, *Cryptosporidium* oocysts are susceptible to heat and desiccation, with greater rates of inactivation expected for oocysts in more arid environments [33]. In the only study that has analyzed the effects of temperature in *Cyclospora* purified oocysts analyzed by microscopy, inactivation was observed at extreme temperatures, such as pasteurization or commercial freezing processes [34], yet were not affected by water chlorination [25,34]. Additionally, traditional spraying practices of some pesticides remain ineffective options of decontamination for farmers [35].

Microscopic examination of sporulated/non-sporulated *C. cayetanensis* oocysts is not feasible in food/fresh produce samples or environmental samples due to the expected presence of low numbers of oocysts in tandem with naturally heterogenous distributions [36,37]. Moreover, enumeration of oocysts is impractical because of excessive amounts of debris in the wash pellets from those samples. In addition to the lack of sensitivity, microscopical analysis of food and environmental samples do not allow for morphological differentiation of the different species of *Cyclospora* which could be present in the environment. Therefore, sensitive and specific molecular methods are needed to analyze such samples, especially at low infection levels. Recently, the US FDA developed a modified real-time PCR method based on a specific mitochondrial target gene (Mit1C qPCR) to detect *C. cayetanensis* in fresh produce. The method was used in single laboratory validation (SLV) studies in Romaine lettuce, cilantro, and raspberries [38] and extended to a multi-laboratory validation (MLV) study in Romaine lettuce [14]. The Mit1C qPCR was shown to be highly sensitive and specific, allowing the detection of as few as five oocysts in the fresh produce inoculated samples (25 or 50 g/sample), and shown to be highly reproducible among laboratories [14,38]. This new real-time PCR marker has also been used to accurately identify the presence of *C. cayetanensis* in inoculated soil samples, using an easy, fast, and sensitive method for the detection of *C. cayetanensis* in soil based on flotation in high density solutions, which was able to detect as few as 10 oocysts in 10 g of soil samples (limit of detection 1 oocyst/g) [37,39].

Despite the importance of the oocyst stage of *Cyclospora* as a major source of human infection, there are few studies concerning the detection of *C. cayetanensis* in contaminated environments. To date, there has been only one previous study analyzing the effects of temperature on *Cyclospora* oocysts in fresh produce [34]. However, that study was performed in experimentally inoculated basil leaves cut into circular pieces of 0.5 cm inoculated with oocysts on the leaf surface inside Eppendorf tubes [34], rather than in actively growing plants. Due to the methodological limitations on *C. cayetanensis* viability studies in food and environmental samples, the present study focused on the molecular detection and persistence of *C. cayetanensis* oocysts in fresh herbs (basil, cilantro and parsley) and in soil artificially contaminated with oocysts of the parasite under different climatic conditions. Herbs were selected based on historically being linked to cyclosporiasis outbreaks in North America, particularly cilantro [3,4,10,40], and basil [3,10,11,13,41,42]. *Cyclospora cayetanensis* contamination was reported in parsley in Egypt [43] and a salad mix of butterhead lettuce, mixed lettuce, dill, parsley, and green onions was associated with an outbreak in salad side dishes in Germany [44].

To our knowledge, there have not been previous studies analyzing environmental conditions and persistence of *C. cayetanensis* oocysts in soil and fresh produce grown under controlled conditions in long-term studies. The main objective of this study was to evaluate the effect of different water contents and temperatures on the detection and persistence of *C. cayetanensis* oocysts in soil and herbs artificially contaminated with the parasite over time. An enhanced understanding of the persistence of *C. cayetanensis* oocysts in herbs and in soil might help target intervention measures for reducing the risk of exposure to oocysts in contaminated fresh produce.

## 2. Materials and Methods

### 2.1. Plants Growth and Oocysts Inoculation in Leaves and Soil

A total of five independent experiments were performed in soil and herbs using a growth chamber CONVIRON^TM^ GEN2000. The daily humidity ranged from 55–65% with a photoperiod of 10 h of sunlight per day in all the experiments. The average time of each experiment was four months, with two of those months needed for the herbs to grow into maturity before inoculation with *C. cayetanensis* oocysts. Experiments 1, 4, and 5 analyzed the effect of arid watering conditions compared to wet watering conditions (experiments 2 and 3). The effect of moderate ambient temperatures (21–25 °C, in daily cycle) (experiments 1 and 2) were compared to elevated temperatures (24–28 °C in a daily cycle) (experiments 3 to 5) (Table 1).

The soil used in the pots was autoclaved prior to the experiments to avoid insect interference, as well as bacterial and fungal growth [37]. In addition, prior to the start of any of the experiments, the soil used was analyzed for the presence of *C. cayetanensis* oocysts or DNA by real-time PCR specific to the parasite [45] and none were detected.

Herbs were initially grown from seeds in 2 seed starter pots (3 inches top diameter: 7.5 cm top diameter (5 cm lower diameter, 6 cm length); 0.233 L volume) (3–4 seeds/pot), with herbs later transferred to large 3-gallon (11.35 L) pots (diameter: 30 cm top diameter and 24 lo diameter, 26 cm height), approximately 5–6 plants per pot. Pots allowed for potential water drainage via 6 drainage holes at the base of the pots over a drainage tray.

Oocysts inoculated were purified *C. cayetanensis* oocysts from a patient in Guatemala, kindly supplied by the Centers for Disease Control and Prevention (CDC). Use of these oocysts was approved by protocol RIHSCID#10-095F. The purified oocysts were processed as previously indicated in [46]. Briefly, after enumeration of several replicates of the concentrated oocyst preparation using a hemocytometer, a dilution of 20 oocysts/µL was prepared in 0.85% sodium chloride for inoculation. No sporulation was observed in the oocyst preparation used for the experiments.

**Figure 1:**
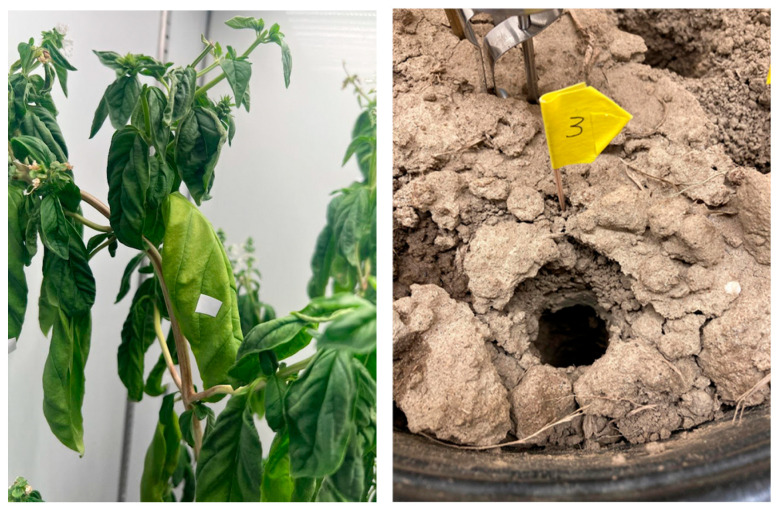
Inoculated leaves with *Cyclospora cayetanensis* oocysts marked with stickers and soil collection sites marked with small, numbered flags for sample collection.

Once the plants were considered mature at roughly eight weeks of growth, the soil was inoculated with 400 *C. cayetanensis* oocysts, adding 20 µL of the solution of 20 oocysts/µL using a pipette and introducing the pipette tip to about 2 cm deep and at eight different spots/experiment. The spots were marked with small, numbered flags near the site where inoculation took place (Figure 1). Individual leaves of the herbs (cilantro, parsley, and basil), on roughly eight different stalks, were inoculated with 100 oocysts at eight different spots each per experiment. Each spot in herb leaves was inoculated with 5 µL in a single droplet of the solution containing 20 oocysts/µL. The leaves inoculated were marked with small white stickers to localize the inoculated leaves in the subsequent sample collections. Samples of soil and leaves were collected at weekly intervals post inoculation for up to 56 days post inoculation (dpi).

Herbs in the arid watering conditions received 500 mL of water once a week while plants in wet conditions received 500 mL of water every other day. Water was measured and applied directly to the soil. Although weekly watering is in accordance with the minimal conditions required for herb survival, improved growth conditions were observed at higher water contents, most probably due to the increased rate of water available to the plants.

Temperatures (ambient) varied from moderate (21–25 °C in a daily cycle) to high (24–28 °C in a daily cycle). The first set of temperatures included the optimal growth temperature for the three herbs which generally prefer an environment in the 18–24 °C range, with 21 °C as the most adequate temperature for growth. The only exception is basil which grows best in temperatures between 24–29 °C. The second set of temperatures included higher temperatures close to the elevated temperature limit for herbs growth conditions, which for the herbs analyzed is around 29 °C.

Initial experiments (1–4) were performed using non-commercial farm soil rich in sand (21.9% clay, 22.8% silt, and 55.3% sand, which after analysis corresponded to Sandy Clay Loam texture). Experiment 5 was performed using another non-commercial agricultural farm soil lower in sand content (46.45 silt, 26.7% clay and 27.0% sand, which after analysis corresponded to a Silt Loam texture) (Table 1). Herbs were expected to grow well in both types of soil, provided they had good drainage.

No soil amendments or fertilizer were added during the experimental periods.

### 2.2. Sample Collection and Processing in Soil and Leaves from Herbs

Soil samples were collected using a Polyvinyl Chloride (PVC) thermoplastic polymer hollow tube collector of 2 cm of diameter and approximately 30 cm of length. The presence of the parasite in soil was confirmed by concentration by flotation in high density sucrose solutions (s.g., 1.12), DNA extraction, and molecular detection by real-time PCR specific for the parasite, as previously indicated [37,39].

The detection of the parasite’s presence in leaves followed the BAM chapter 19b method, with three main steps including washing and concentration of the parasite, DNA extraction, and molecular detection by real-time PCR [45]. Briefly, the wash protocol to recover the oocysts from fresh produce was performed using 50 mL of 0.1% Alconox^®^ detergent and sequential centrifugations/aspirations to recover, pool, and concentrate the wash debris. After this step, DNA isolation was performed using the FastDNA SPIN Kit for Soil in conjunction with a FastPrep-24 Instrument (MP Biomedicals, Santa Ana, CA, USA) for both soil and herbs.

Amplification and quantitation of *C. cayetanensis* specific DNA was performed as previously described using real-time PCR in a duplex reaction, targeting both the specific *C. cayetanensis Cox3* gene located in the mitochondrial genome (Mit1C target) and an exogenous internal amplification control [14,38]. Real-time PCR was performed in an Applied Biosystems 7500 Fast Real-Time PCR System (ThermoFisher Scientific, Waltham, MA, USA). The commercially prepared synthetic gBlocks gene fragment Mit1AA (Integrated DNA Technologies, Coralville, CA, USA) was used as a positive control for amplification of the *C. cayetanensis* Mit1C gene. Serial dilutions of the positive control target covering three orders of magnitude ranging from 5 × 10^2^ to 5 copies/μL were prepared. A volume of 2.0 μL DNA of the appropriate samples and positive control dilutions were used as template in real-time PCR reactions. Each experimental real-time PCR run consisted of study samples, a non-template control (NTC), and positive controls containing 10-fold serial dilutions from 10^3^ to 10 copies of the Mit1AA DNA control analyzed in triplicate reactions. Runs were only considered valid if all three replicates of the positive control reactions produced the expected positive result with a cycle threshold (C_T_) 38.0 or lower. Samples were considered positive if one or more replicates produced *C. cayetanensis* target reaction with a C_T_ value ≤ 38.0 for the Mit1C target [14,38]. Reactions with C_T_ values greater than 38.0 were considered negative and considered inconclusive due to inhibition if the IAC reaction failed or produced an average C_T_ value more than three cycles higher when compared to the NTC [14,38]. Data were analyzed qualitatively, and results were considered positive or negative.

## 3. Results

### 3.1. Experiments in Sandy Clay Loam (SCL)

Experiments 1 to 4 were performed using the same SCL farm soil samples as previously described. On a few occasions at the end of the experimental period at 56 dpi, leaves from one or multiple of the three herbs were not available for sampling collection. At that time, some of the leaves were found to have fallen during the experimental period or to be completely desiccated

**Figure 2:**
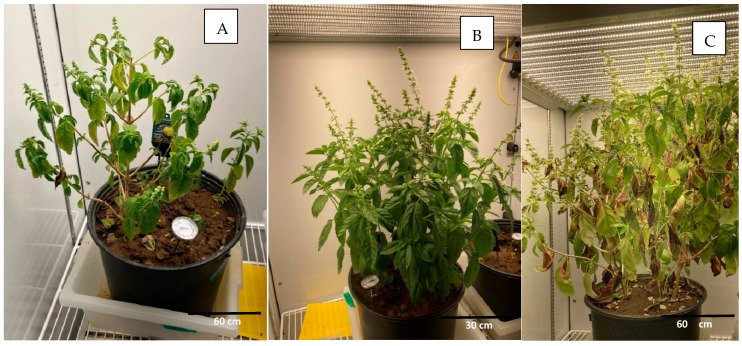
Comparison of growth of basil under controlled conditions in growth chambers inoculated with *Cyclospora cayetanensis* oocysts in (**A**)) arid conditions under moderate temperature daily cycle (21–25 °C) at 42 dpi (experiment 1), (**A**) and in wet conditions ((**B**): experiment 2 and C: experiment 3). (**B**) Basil growth in wet conditions under moderate temperature daily cycle (21–25 °C) at 42 dpi (experiment 2), and (**C**) basil growth in wet conditions at high temperature (24–28 °C) at 42 dpi (experiment 3).

In experiment 1, the effect of temperatures ranging from 21–25 °C in a daily cycle in the CONVIRON 2000^TM^ growth chambers (Conviron USA, Pembina, ND, USA) were analyzed, mimicking moderate/high temperatures in arid water conditions (Table 1). The herb plants, started as seeds, grew slowly under these conditions and were relatively small in the arid conditions in SCL texture (Figure 2A). The most affected herb growth in the arid conditions was parsley, germinating the slowest and expectedly yielding the lowest numbers of seedlings.

In the collected herb leaves, the presence of the parasite was observed in the three analyzed herbs (basil, parsley, and cilantro) at every collection time. Average C_T_ values varied among samplings, ranging from 33.20 ± 1.47 to 37.38 ± 3.57, with exception of the last collection time, when no leaves of the individual herbs were available for collection (Table 2). On the other hand, the presence of the parasite in soil was confirmed by real-time PCR specific for the parasite only at samples collected at 7 dpi (Average C_T_ values of 34.37 ± 1.86) (Table 3), with sample collections from 14 dpi–56 dpi all being negative for the presence of the parasite in soil (Table 3). No inhibition was observed in any of the soil samples analyzed.

In experiments 2 and 3, in wet conditions in SCL soil, with waterings taking place every two–three days, the parasite was detected in all leaf sample collections during the study, independent of temperature (experiment 2 and 3) (Table 2). Similarly, the parasite was detected in all soil samples collected throughout the study period (Table 3), potentially indicating biologically available soil moisture under those experimental conditions.

**Figure 3:**
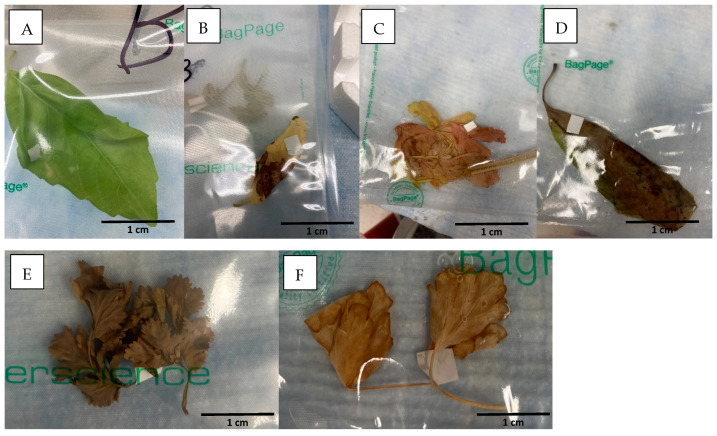
Examples of collected leaves to be processed inside their processing filter-bags. (**A**) Normal green leaf, (**B**,**C**) wilted leaves; (**D**) mostly dried leaf; (**E**,**F**) completely dried leaves.

In experiments 2 and 3, under wet conditions, with increased water availability, the herbs exhibited greater growth compared to that observed in experiment 1 (Figure 2B,C). Growth was particularly large in basil in wet conditions and at high temperatures (experiment 3) (Figure 2C). On the other hand, at higher temperatures, some leaves experienced leaf desiccation and wilting (yellowing and brown discoloration), particularly in samples collected 42–52 dpi and onwards (Figure 2C and Figure 3). In those climatic conditions, both wilted and non-wilted leaves of cilantro were collected at 21 dpi in experiment 3 (Table 4, Figure 3). Both types of leaves were found positive to the presence of the parasite in similar average C_T_ values (32.7 in normal leaf versus 31.7 in wilted leaf). As such, this seems to indicate that the wilted leaves had enough moisture content to maintain oocyst integrity when collected and analyzed.

Only a sample of soil collected at 56 dpi in experiment 2 was found to have inhibition based on the IAC C_T_ values. This inhibited soil sample DNA was cleaned up using a commercial kit (OneStep^TM^ PCR inhibitor removal, Zymo Research, Irvine, CA, USA) and the replicates of this soil sample were found to be undetermined after the clean-up after a new real-time PCR assay was run. A soil sample on experiment 3 collected at 35–38 dpi was the only other soil sample found negative for the presence of the parasite.

Experiment 4 analyzed the effect of hot temperatures (24 °C–28 °C) in arid watering conditions in SCL soil. The three herbs, basil, cilantro, and parsley, grew slowly and showed wilted and dried leaves as early as four weeks post inoculation, with the presence of wilted leaves increasing along the study period. Occasionally the leaf collected was wilted and/or partially dried. However, the presence of the parasite was observed in those wilted and partially dried leaves (Table 4). At every collection time, the presence of the parasite was observed in the three analyzed herbs (basil, parsley, and cilantro) (Table 2), and even wilted leaves showed average C_T_ values of *C. cayetanensis* similar among sampling periods (Table 4). Only when leaves were completely dried (Figure 3) was *C. cayetanensis* detection negative, as was observed in the last sample collection in this experiment, in cilantro and parsley. On the other hand, as observed in arid conditions at moderate temperature in experiment 1, the presence of the parasite in soil was only confirmed by real-time PCR at samples collected at 7 dpi and 14 dpi (Table 3) while the rest of the soil collection dates were negative for the presence of the parasite in soil (Table 3). No inhibition was observed in any of the soil samples analyzed in experiment 4.

#### Analysis of Presence of Oocysts in Leachate Water in Experiment 2 in Sandy Clay Loam

Leachate and residual water after watering of herbs, was not observed in arid watering conditions (500 mL of water used per watering in a week), but some spill/leachate water was observed in the trays underneath the pots on a few occasions in experiment 2 under wet watering conditions (500 mL of water used per watering every other day) and moderate temperatures. When that residual water/sediment was observed in the trays located at the base of the pots after watering (only in two occasions), the residual water/sediment was collected and analyzed via sucrose flotation way as described for soil processing. Parasite DNA was not found, indicating no *Cyclospora* oocyst translocation through the soil.

### 3.2. Experiment on Silt Loam Soil (Experiment 5)

An experiment in Silt Loam soil was performed in the most strenuous conditions of the previous experiments in SCL soil (high temperature and arid watering conditions) to study the possible effect of the texture of the soil on the detection of the parasite.

**Figure 4:**
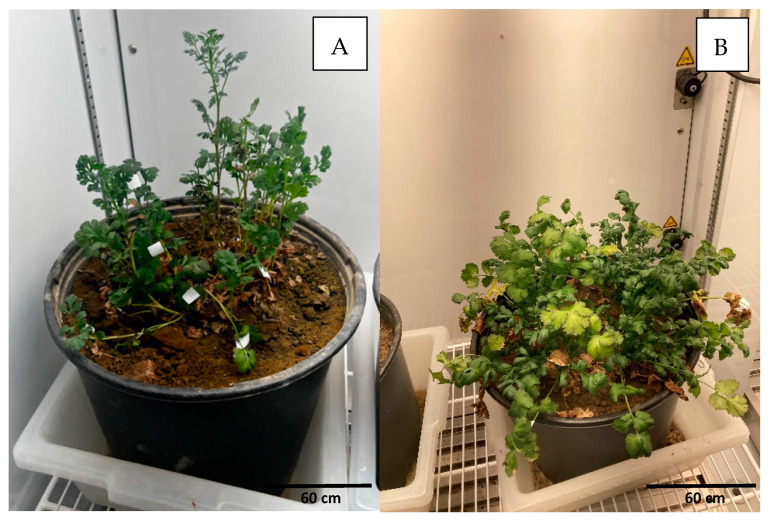
Small leaves in cilantro growing under arid watering conditions in Silt Loam soil (**A**) compared to more normal leaves in Sandy Clay Loam (**B**) under the same conditions at 42 dpi.

In Silt Loam soil under high temperature and arid conditions, herbs grew slowly, as had been also observed in experiment 4 in SCL soil. Cilantro was the herb which showed slower growth and produced tinier leaves (Figure 4). The presence of the parasite was observed in the three analyzed herbs (basil, parsley, and cilantro) at every collection time with exception of the last collection time when no basil leaf was available and the other two leaves (cilantro and parsley) were found completely desiccated (Table 5). On the other hand, the parasite detection in Silt Loam texture was only observed up to 21 dpi, with negative results observed after that date collection (Table 5), consistent with previous experiments.

**Figure 5:**
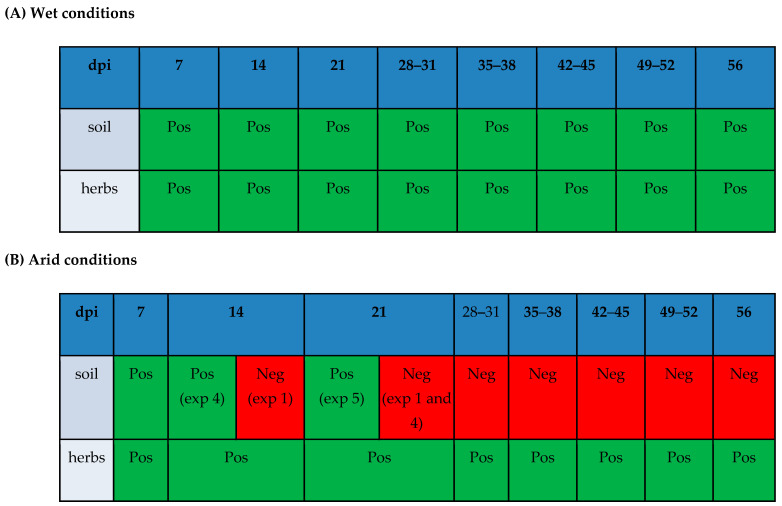
Graphic visualization of results in soil and herbs grown in wet watering conditions ((**A**) experiments 2 and 3) versus in soil and herbs grown in arid watering conditions ((**B**) experiments 1 and 4 in Sandy Clay Loam soil, and experiment 5 in Silt Loam soil). When different results were observed among experiments in the same dpi collection, the experiment is indicated in parenthesis. Pos: positive, Neg: negative, exp.: experiment.

A graphic visualization of results in soil and herbs grown in wet conditions (A: experiments 2 and 3) versus in soil and herbs grown in arid conditions (B: experiments 1 and 4 in Sandy Clay Loam soil, and experiment 5 in Silt Loam soil) is shown in Figure 5.

## 4. Discussion

In this study the presence and persistence of *C. cayetanensis* oocysts in artificially contaminated herbs (basil, cilantro, and parsley) and soil was analyzed in different environmental conditions using growth chambers. To our knowledge, this is the first study on the detection of *C. cayetanensis* in soil and plants growing under controlled climatic conditions in long-term studies (up to 56 days post inoculation). Ultimately, our study serves to expand the literature gap on *C. cayetanensis* persistence in the environment to better understand the effect of different climatic conditions, which could help in intervention measures for reducing the risk of exposure to oocysts in contaminated fresh produce.

Cyclosporiasis is a foodborne illness related to the consumption of contaminated fresh produce, which are consumed without prior processing to inactivate or remove the oocysts [3,47,48,49]. Pasteurized foods or foods thoroughly heated before consumption have not been associated with illness [19]. However, there are still significant gaps in our knowledge of the epidemiology of *C. cayetanensis*, including the contribution of contaminated soil as a source of food contamination. Contact with soil could play a role in the contamination of foods and has been included as a risk factor for *C. cayetanensis* infection in several studies [50,51,52,53]. Therefore, it is important to ascertain the persistence of the parasite in soil as well as in fresh produce. In the present study, the detection and persistence of the inoculated *C. cayetanensis* oocysts in herb plants and soil were evaluated in several experiments, using a specific and highly sensitive real-time PCR. Unfortunately, biologically active or inactive oocysts cannot be differentiated, as is the case for any molecular method for detection based on DNA. Therefore, the results provide the maximum occurrence and levels of contamination for a given matrix, rather than an indication of the metabolically active state of *Cyclospora* oocysts [54]. As was observed in previous studies [37,39], the protocol used for the detection of *C. cayetanensis* in soil was not prone to inhibition. Only a sample of soil collected at 56 dpi in experiment 2 was found to have inhibition based on the IAC C_T_ values.

Remarkably, independent of the studied temperature (moderate/hot), experimental water conditions (arid or wet conditions), and soil texture (Sandy Clay Loam and Silt Loam), the presence of the parasite was observed in leaves collected from the three herbs during the whole experimental period in every experiment. Protozoa oocysts can survive for several months in water and moist environments [28,29]. Nevertheless, moisture and relative humidity have been reported as crucial in the maintenance of parasites in the environment [26], and in food, optimal moisture has been found critical for maintaining the vitality of *Cryptosporidium* and *Giardia* oocysts until they are ready for the next stage in their life cycle [26]. One of the few studies that have described the climate effect on *Cyclospora* spp. was done in field conditions in a semiarid region in Venezuela. This study indicated that *Cyclospora* infections predominated in the months of higher rainfall, which supports the idea that mean annual rainfall and the consequent moisture of the soil aided in the survival and maintenance of *Cyclospora* spp. oocysts in the soil [55]. In the present study, the persistence of the parasite DNA was observed even in wilted and partially dried leaves, most probably due to the available residual moisture in those leaves. On the other hand, we found that completely desiccated and dried leaves were negative for the detection of the parasite. These results further emphasize that the potential residual moisture in herb leaves was the main factor affecting detectability and persistence of *C. cayetanensis* in the herbs, even under arid conditions in the experiments.

It was interesting to observe that the range of temperatures analyzed in the study did not influence the presence or detectability of the parasite in herbs under both wet and arid water conditions, especially since some studies found temperature and not moisture to be limiting factors. Although high temperatures (more than 35 °C) are deleterious for *T. gondii* oocysts [30], *Cryptosporidium* spp., [56] or even *C. cayetanensis* [19,34], those temperatures ranges do not fall within the suitable temperature range for the growth of the fresh produce in the present study. Within temperate climates, *T. gondii* and *Cryptosporidium* spp. oocysts are known to persist and remain viable for months in agricultural regions [28,57,58,59]. The results of the present study in herbs under controlled conditions agree with those previous results, meaning that temperature is most likely not a contributing factor for *Cyclospora* persistence if there is enough water or residual moisture in the agricultural environment.

Similarly to the detection on the leaves, under wet conditions and under both moderate and hot temperature regimes, the parasite was detected also in the soil at each sampling collection time. There was an exception in experiment 3 (under wet conditions and high temperatures) at 35–38 dpi, where the collected sample tested negative for the presence of parasite DNA. However, the parasite was found in the soil collected at previous and subsequent time points in the same experimental conditions. Therefore, the most probable explanation was the loss of parasite DNA during the processing of that soil sample collection. The long persistence of the parasite in soil, under wet conditions, again indicates that if there was enough water content present in the soil, the parasite will persist at the range of temperatures analyzed in the study, as observed with other protozoa [33].

The main differences on detection were observed in the soil in experiments under arid conditions, in which the parasite showed short-lived persistence in the soil. In the present study, there was no indication that the parasite oocysts percolated outside the pots during the experimental watering conditions. During arid conditions, no water was observed to spill into the trays under the pots. Only on a few occasions in wet conditions was residual water/sediment observed in the collection trays, but those water/sediments tested negative for the parasite. Therefore, these results indicate that the negativity of soil samples to the presence of the parasite in arid conditions was not a result of oocyst loss during the watering process in the experiments. Soil aridity has been shown to reduce the viability of protozoa, such as *T. gondii*, *Giardia*, and *Cryptosporidium* spp. in soil [23,30,60,61]. Oocysts are vulnerable to dryness and desiccation, with desiccation being a well-known way to kill oocysts of other protozoa [33,62,63]. Extreme dryness in the southwestern US was suspected to be the cause of decrease of the survival time of *T. gondii* oocysts in the soil, although this did not eliminate all the viable oocysts [64]. Similarly, in some areas of California where temperatures frequently reach 30 °C and have low humidity, especially during the summer, *T. gondii* was not detected in soil [59]. In some studies, however, soil moisture in the ranges tested was not shown to be influential to oocyst survival [56,57,65]. In contrast, our study revealed a different outcome. We found that soil desiccation adversely affects the presence of *C. cayetanensis* oocysts in the soil. The short-lived persistence in the soil under arid conditions was consistent, regardless of the temperature (ranging from moderate to hot, suitable for herb plant growth) and soil texture. Specifically, samples tested positive for *C. cayetanensis* for a maximum of 14 dpi in Sandy Clay Loam and up to 21 dpi in Silt Loam. Some previous studies reported an effect of soil texture in the detection/recovery of the parasite in soil, with lower *T. gondii* detection in soil with high proportions of sand [36,57,58,66]. It was reported that when incubated at 20 °C, *Cryptosporidium parvum* oocyst exhibited better survival rates in the Silt Loam soil than in a Silty Clay Loam or loamy sand. In contrast, at 30 °C, the oocyst showed a significantly lower survival rate in the Silt Clay Loam than compared to the other two soil types [57]. The increased diameter of sand grains would contribute to greater overall macro-porosity compared to other textures, resulting in greater rates of water drainage as compared to finer textures like Silt Loams [67]. In the present study, slightly longer persistence was observed in Silt Loam, but the two main textures observed in farm soils analyzed in the study showed comparable results in terms of short-term persistence under arid conditions. Differing susceptibilities of some protozoa species oocysts to desiccation have been observed, for example among *Eimeria* species [68]. Additionally, it has been described that *Isospora* species in birds have evolved inheritance adaptive traits that allow the release of oocysts in the late afternoon to avoid desiccation and U.V. radiation, thus reducing mortality of the oocysts in the external environment [69]. Inheritance adaptive traits or different susceptibility among *Cyclospora* species to climatic factors are currently unknown.

## 5. Conclusions

In conclusion, in wet conditions, the parasite demonstrated long-term persistence of oocysts in soil and in herbs when herbs are grown at moderate or high temperatures. In contrast, in arid conditions, the parasite demonstrated only short-lived persistence in the soil, while it persisted in the leaves throughout the experimental period independently of temperature variations. These results confirm that *Cyclospora* oocysts are highly resistant in the environment, indicating that in normal herb-growing conditions, the parasite will persist in leaves during the growing season. This makes it challenging to implement effective control measures to interrupt the parasitic life cycle in areas where produce is grown. This highlights the importance of control measures in and near growers’ fields, such as access to toilet and handwashing facilities, correct management of septic systems and portable toilets on the farm, thorough hand washing, and the proper disposal and treatment of human sewage. Additionally, workers having any symptoms of gastro-enteritis should not be allowed contact with vegetables or food areas [3,15]. Recent studies have shown that interventions to reduce *Eimeria*, as a surrogate parasite for *C. cayetanensis*, in irrigation water using filtering systems may be able to reduce fresh produce contamination with the parasite [70,71]. Our results on the effect of desiccation on the presence and persistence of oocysts may provide useful insights for the proper cleaning and sanitizing of utensils or food contact surfaces, such as harvesting equipment to help control the persistence of the parasite. Further studies would need to be performed to verify this hypothesis.

## Figures and Tables

**Table 1:** Experimental conditions for soil and herbs (basil, cilantro, and parsley) inoculated with *C. cayetanensis* oocysts in growth chambers.

Experiment No.	Water Conditions	Temperature Conditions	Type of Soil
Experiment 1	Arid (watering once a week)	Moderate (21–25 °C)	Sandy Clay Loam
Experiment 2	Wet (watering every 2–3 days)	Moderate (21–25 °C)	Sandy Clay Loam
Experiment 3	Wet (watering every 2–3 days)	High (24–28 °C)	Sandy Clay Loam
Experiment 4	Arid (watering once a week)	High (24–28° C)	Sandy Clay Loam
Experiment 5	Arid (watering once a week)	High (24–28° C)	Silt Loam

**Table 2:** Average C_T_ values in leaves of herbs (combined results basil, cilantro, and parsley findings) sampled at each collection day after inoculation with *Cyclospora cayetanensis* oocysts in Sandy Clay Loam soil.

	Experiment 1	Experiment 2	Experiment 3	Experiment 4
Collection Day	Average C_T_ ± Stdev	Average C_T_ ± Stdev	Average C_T_ ± Stdev	Average C_T_ ± Stdev
7 dpi	33.24 ± 1.47	32.45 ± 0.76	33.73 ± 1.76	34.52 ± 1.71
14 dpi	34.41 ± 0.97	37.07 ± 0.52	35.67 ± 1.63	30.49 ± 0.06
21 dpi	35.06 ± 1.37	34.37 ± 1.38	33.25 ± 0.55	33.06 ± 0.46
28–31 dpi	33.93 ± 1.22	32.86 ± 0.34	34.02 ± 2.03	34.65 ± 1.20
35–38 dpi	33.70 ± 0.56	33.34 ± 0.21	32.88 ± 0.85	35.23 ± 2.57
42–45 dpi	37.38 ± 3.57	35.39 ± 1.14	33.30 ± 1.85	35.73 ± 0.65
49–52 dpi	33.58 ± 0.39	35.38 ± 1.40	33.60 ± N/A **#	35.88 ± 0.50
56 dpi	N/A *	35.62 ± 1.41	33.70 ± N/A **#	37.62 ± N/A **&

N/A: Not available; * No leaves collected at that time point; ** Only one type of leaf collected was found to be positive; # Only cilantro positive; & Only basil positive.

**Table 3:** Average C_T_ values in soil collected in four experiments in Sandy Clay Loam texture.

	Experiment 1	Experiment 2	Experiment 3	Experiment 4
Collection Day	Average C_T_ ± Stdev	Average C_T_ ± Stdev	Average C_T_ ± Stdev	Average C_T_ ± Stdev
7 dpi	34.47 ± 1.86	31.67 ± 1.02	38.0 ± N/A	36.4 ± 0.09
14 dpi	und	32.43 ± 0.37	36.6 ± N/A	37.44 ± 0.76
21 dpi	und	29.57 ± 0.56	37.5 ± N/A	und
28–31 dpi	und	33.74 ± N/A	35.15 ± 0.73	und
35–38 dpi	und	35.43 ± N/A	und **	und
42–45 dpi	und	35.62 ± 0.93	35.33 ± 1.30	und
49–52 dpi	und	35.99 ± 1.33	31.7 ± 0.50	und
56 dpi	und	und *	30.4 ± 0.26	und

und: Undetermined; N/A: Not available (only one sample replicate positive in real-time PCR); * Soil sample was inhibited; ** Negative in wet conditions.

**Table 4:** Average C_T_ values in individual cilantro herb leaves as an example (wilted, dried, or normal) in experiments 3 and 4 conducted at high temperatures (see pictures).

Experiment	Condition of Leaf Collected	Average C_T_ Values and Standard Deviation (3 Replicates)
Experiment 3	Normal leaf 21 dpi	32.7 ± 1.2
Experiment 3	Wilted leaf 21 dpi	31.7 ± 0.13
Experiment 4	Normal leaf 31 dpi	35.5 ± 1.5
Experiment 4	Mostly dried leaf 38 dpi	32.3 ± 0.5
Experiment 4	Mostly dried leaf 45 dpi	35.1 ± 1.1
Experiment 4	Mostly dried leaf 52 dpi	35.5 ± 1.7
Experiment 4	Completely dried leaf 56 dpi	und

und: undetermined.

**Table 5:** Comparison of result in leaves in Sandy Clay Loam and Silt Loam soil in same conditions of water and temperature (high temperature and arid watering conditions).

	Leaves		Soil	
	Sandy Clay Loam Soil Experiment 4	Silt Loam Soil Experiment 5	Sandy Clay Loam Soil Experiment 4	Silt Loam Soil Experiment 5
Collection Day	Average C_T_ ± Stdev	Average C_T_ ± Stdev	Average C_T_ ± Stdev	Average C_T_ ± Stdev
7 dpi	34.52 ± 1.71	35.98 ± 0.61	36.4 ± 0.09	35.90 ± 0.31
14 dpi	30.49 ± 0.06	36.95 ± 0.12	37.44 ± 0.76	36.44 ± 0.45
21 dpi	33.06 ± 0.46	36.76 ± 1.46	und	36.56 ± 1.17
28–31 dpi	34.65 ± 1.20	34.88 ± 0.21	und	und
35–38 dpi	35.23 ± 2.57	36.35 ± 1.28	und	und
42–45 dpi	35.73 ± 0.65	37.24 ± 1.07	und	und
49–52 dpi	35.88 ± 0.50	37.56 ± N/A	und	und
56 dpi	37.62 ± N/A	N/A *	und	und

* No leaf collected at that time point from basil. Complete desiccation of cilantro and parsley leaves. und: undetermined. N/A: Not available (only one sample replicate positive in real-time PCR).

## Data Availability

The data used to support the findings are including within the article; further inquiries can be directed to the corresponding author.
